# Prevalence and associations of metabolic syndrome in patients with alcohol use disorder

**DOI:** 10.1038/s41598-022-06010-3

**Published:** 2022-02-16

**Authors:** Anna Hernández-Rubio, Arantza Sanvisens, Ferran Bolao, Isabel Cachón-Suárez, Carme Garcia-Martín, Antoni Short, Ramón Bataller, Roberto Muga

**Affiliations:** 1 Department of Internal Medicine, Hospital Universitari Germans Trias i Pujol-IGTP, 08916 Badalona, Spain; 2 Department of Medicine, Universitat Autònoma de Barcelona, Barcelona, Spain; 3 Girona Cancer Registry, Institut Català d’Oncologia, Girona, Spain; 4 Department of Internal Medicine. Hospital, Universitari de Bellvitge-IDIBELL, L’Hospitalet de Llobregat, Barcelona, Spain; 5 Department of Clinical Analysis and Biochemistry, Laboratori Clinic Metropolitana Sud, Hospital Universitari de Bellvitge-IDIBELL, L’Hospitalet de Llobregat, Barcelona, Spain; 6 Department of Clinical Analysis and Biochemistry, Laboratori Clinic Metropolitana Nord, Hospital Universitari Germans Trias I Pujol, Badalona, Spain; 7 Alcohol Unit, Hospital Universitari Son Espases - IdISPa, Palma de Mallorca, Spain; 8 Division of Gastroenterology, Hepatology, and Nutrition, Department of Medicine, Center for Liver Diseases, Pittsburgh Liver Research Center, University of Pittsburgh, Pittsburgh, PA USA

**Keywords:** Endocrine system and metabolic diseases, Metabolic disorders, Addiction

## Abstract

Excessive alcohol consumption has been associated with different components of the metabolic syndrome (MetS) such as arterial hypertension, dyslipidemia, type 2 diabetes or obesity. We aimed to analyze the prevalence and associations of MetS in patients with Alcohol Use Disorder (AUD). Cross-sectional study in heavy drinkers admitted for the treatment of AUD between 2013 and 2017. Medical comorbidity, anthropometric data, alcohol use and biological parameters were obtained. MetS was established according to the harmonized definition. A total of 728 patients (22% women) were included; median age was 47 years (IQR: 40–53.5), median alcohol consumption was 160 g/day (IQR: 115–240) and prevalence of MetS was 13.9%. The multivariate analysis showed a significant dose–response effect of estimated glomerular filtration (eGFR) and MetS: relative to patients with eGFR > 90 mL/min, those with eGFR (60–90 mL/min) and those with eGFR < 60 mL/min were 1.93 times (95% CI 1.18–3.15) and 5.61 times (95% CI 1.66–19.0) more likely to have MetS, respectively. MetS was significantly associated with hyperuricemia (OR 2.28, 95% CI 1.36–3.82) and elevated serum GGT (OR 3.67, 95% CI 1.80–7.46). Furthermore, for every increase of 1 year in age, the probability of MetS increased significantly (OR 1.03, 95% CI 1.01–1.05). MetS in heavy drinkers is independently associated with reduced kidney function and metabolic risk factors including hyperuricemia and elevated serum GGT.

## Introduction

The metabolic syndrome (MetS) is a cluster of clinical and biological findings including obesity, hypertension, glucose intolerance, and dyslipidemia, which are related to each other and confer a higher risk of cardiovascular disease (CVD) and type 2 diabetes^1^. The prevalence of MetS is increasing in western countries and it is estimated to affect almost a quarter of the general European population^2,3^.

In 1998, the World Health Organization defined MetS as the presence of glucose intolerance, low level of HDL cholesterol, high triglycerides, obesity, and/or hypertension^4^. Subsequent modifications were introduced and several definitions appeared within a decade^5–7^. In 2009, the American Heart Association and National Heart, Lung and Blood Institute, International Diabetes Federation, World Heart Federation, International Atherosclerosis Society, and International Association for the Study of Obesity established a harmonized definition that have been maintained to date^8^. However, some authors proposed the use of apolipoprotein B to better identify individuals with atherogenic dyslipidemia^9^. Moreover, some studies focus on characterizing premorbid MetS, since a proportion of individuals do not develop type 2 diabetes or CVD in the initial stages of the syndrome^10,11^. In clinical practice, the identification of individuals with premorbid MetS can be useful by revealing a patient phenotype that would benefit from pharmacologic and non-pharmacologic interventions to avoid the incidence of cardiometabolic complications.

Five percent of the population aged 15 to 64 years presented a pattern of at-risk alcohol consumption in Spain and alcohol was responsible for 35% of treatment admissions in addiction units^12^. Furthermore, one in five people with alcohol use disorder (AUD) have MetS and studies have identified geographic differences in relation with life styles and environmental factors^13^ due to arterial hypertension and dyslipidemia, among other mechanisms^14,15^. Moreover, unhealthy lifestyles (i.e., sedentarism, tobacco smoking) may be relatively frequent in individuals with alcohol misuse. In fact, CVD is the leading cause of death in patients with AUD^16^. In addition, the incidence of type 2 diabetes in heavy drinkers may be higher than observed in the general population^17^. On the other hand, the relationship between excessive alcohol consumption and overweight is controversial, and the association between the amount and type of alcohol with obesity has not been fully established. Nevertheless, cardiometabolic factors and MetS have been poorly studied in heavy drinkers as compared to other populations. We hypothesize that chronic, excessive alcohol consumption favors the appearance of pro-inflammatory alterations that predisposes to both type 2 diabetes and atherosclerotic CVD. Moreover, co-occurrence of liver damage (i.e., alcoholic steatohepatitis) in the context of excessive alcohol consumption can contribute to the inflammatory response thus increasing the cardiometabolic risk. The objective of this study was to analyze the impact and associations of MetS in heavy drinkers seeking treatment of alcohol abuse or dependence.

## Methods

This was a cross-sectional study in patients admitted to two hospital addiction units in metropolitan Barcelona, Spain: Hospital Universitari de Bellvitge in L’Hospitalet de Llobregat and Hospital Universitari Germans Trias i Pujol in Badalona. All patients had been consecutively admitted between January 2013 and November 2017 and were referred by primary care physicians and specialists in Addiction medicine at outpatient clinics. All patients were diagnosed with AUD according to the Diagnostic and Statistical Manual of Mental Disorders, 5th edition.

On the day of admission, patients underwent an interview to collect data regarding sociodemographic variables, medical comorbidity (i.e., type 2 diabetes, hypertension), antecedent of CVD (i.e., coronary artery disease, ischemic stroke, peripheral vascular disease), and characteristics of alcohol consumption (i.e., daily amount consumed, age of starting alcohol consumption, duration of AUD, previous treatment of the disorder); alcohol consumption was quantified as grams of ethanol per day. Anthropometric data (height and weight) were also recorded.

Peripheral blood samples for determining biochemical and hematological parameters were collected on the second day of admission after overnight fasting. Blood samples were collected in heparin plasma tubes and transferred to the laboratories. Hematological parameters, such as hemoglobin, erythrocyte sedimentation rate (ESR), mean corpuscular volume, and serum folate, were measured using automated hematological analyzers. Biochemical parameters, such as glucose, urate, albumin, total cholesterol, HDL cholesterol, triglycerides, total bilirubin, creatinine, uric acid, aspartate aminotransferase, alanine aminotransferase (ALT), and gamma-glutamyl transferase (GGT), were measured with standard enzymatic methods using multichannel automatic analyzers. The analytical alterations were defined based on the reference values determined by the laboratories. The established cut-off value for serum uric acid (SUA) was > 7.2 mg/dL in men and > 6 mg dL in women. High levels were defined as > 1.2 mg/dL for creatinine, > 50 U/L for GGT, and > 41 U/L for ALT. Altered ESR was defined as > 20 mm. The clinical chemistry and hematology laboratories complied with the UNE-EN-ISO9001:2015 standards and were accredited in their respective areas.

Renal function was assessed by the estimated Glomerular Filtration Rate (eGFR), calculated using creatinine, age, sex, and ethnicity through the Chronic Kidney Disease Epidemiology Collaboration equation^18^. Highly reduced and mildly reduced eGFR values were defined as ≤ 60 mL/min/1.73 m^2^ and 60–90 mL/min/1.73 m^2^, respectively.

Pharmacologic treatment during admission included benzodiazepines, vitamin B complex, and other pharmacotherapy depending on the severity of alcohol withdrawal.

The length of hospital stay was 7 days on average, and patients were advised to follow visit and control protocols at the outpatient clinic after discharge.

### Metabolic syndrome

MetS was defined according to the diagnostic criteria of 2009^8^ that required the presence of three or more of the following: (1) central obesity; (2) triglycerides ≥ 150 mg/dL or treatment with fibrates, nicotinic acid, or ω-3 fatty acids; (3) HDL cholesterol < 40 mg/dL in men and < 50 mg/dL in women or treatment with fibrates or nicotinic acid; (4) systolic blood pressure ≥ 130 mmHg and/or diastolic blood pressure ≥ 85 mmHg or antihypertensive treatment; and (5) elevated fasting glucose ≥ 100 mg/dL or treatment for type 2 diabetes. For the purpose of this study, central obesity was assumed if the body mass index (BMI) at admission was > 30 kg/m^2^^19,20^. Moreover, atherogenic dyslipidemia was defined as the simultaneous alteration of elevated triglycerides (> 150 mg/dL) and low HDL cholesterol (< 40 mg/dL in men and < 50 mg/dL in women).

### Selection criteria

There were 949 consecutive admissions of 739 patients during the study period, with 99% of caucasian origin. For those patients who were admitted more than once, the first admission was selected. Eleven patients were excluded due to insufficient information to determine MetS.

### Ethical considerations

All patients provided written informed consent, and the study design was approved by the Ethics Committee of the Hospital Universitari Germans Trias i Pujol (approval number CEXT042013). The methods complied with the ethical standards for medical research and the principles of good clinical practice in accordance with the World Medical Association’s Declaration of Helsinki.

### Statistical analysis

Descriptive statistics were expressed as median (interquartile range [IQR]) for quantitative variables and as absolute frequencies and percentages for qualitative variables. To analyze differences in those with and without MetS we used the chi-square test for qualitative variables and the t-test for analyzing mean differences in the quantitative variables.

Logistic regression models were used to analyze associations of alcohol use characteristics and blood parameter alterations with MetS. The covariates included in the multivariate analysis were those that were found to be statistically significant in univariate analysis. The Hosmer–Lemeshow test was used for analyzing goodness of fit.

A sensitivity analysis was performed with a cut-off point of > 25 kg/m^2^ for BMI as a MetS criteria.

*P* values < 0.05 were considered statistically significant. Statistical analyses were performed using Stata software (version 11.1, College Station, Texas, USA).

## Results

This study included 728 patients (78% men) with a median age of 47 years (IQR: 40–53.5 years) who started drinking alcohol at 16 years (IQR: 16–18 years). Among them, the median alcohol consumption was 160 g/day (IQR: 115–240 g/day). Moreover, 92.5% of the patients were or had been smokers and 14.4% were current cocaine users.

Table 1 shows the sociodemographic, alcohol use characteristics and blood parameters of patients at admission.

**Table 1:** Characteristics of 728 patients with and without Metabolic Syndrome at admission to treatment of AUD.

	N = 728 n (%)	No MetS N = 627 n (%)	MetS N = 101 n (%)	*P* value
***Sociodemographic***				
Women	160 (22.0)	137 (21.8)	23 (22.8)	0.835
Age at admission, *median (IQR)*	47 [40–53.5]	46 [40–53]	51 [45–55]	** < 0.001**
***Alcohol-related variables***				
Age at starting alcohol use, *median (IQR)* (n = 673)	16 [16–18]	16 [16–18]	16 [16–20]	0.223
Antecedent of alcohol treatment (n = 673)	490 (72.8)	422 (72.9)	68 (72.3)	0.912
Amount of alcohol consumption (g/day), *median (IQR)* (n = 678)	160 [115–240]	160 [110–240]	180 [120–250]	0.113
***Biological parameters and prevalence of alterations***				
Hemoglobin (g/dL) (n = 726), median [IQR] < 13 g/dL men; < 12 g/dL women	14.3 [13.2–15.4]	14.3 [13.2–15.4]	14.5 [13.3–15.6]	0.43
	95 (13.1)	85 (13.6)	10 (9.9)	0.306
ESR (mm) (n = 687), median [IQR] > 20 (s)	8 [4–18]	8 [4–17]	10 [6–24]	**0.001**
	148 (21.5)	120 (20.4)	28 (28.6)	0.068
MCV (fl) (n = 717), median [IQR] > 100 (fl)	96 [92.1–100.4]	96 [92.2–101]	95 [92–100]	0.238
	183 (25.5)	163 (26.5)	20 (19.8)	0.155
Fibrinogen (g/L) (n = 689), median [IQR] > 4.5 (g/L)	3.1 [2.6–3.9]	3.1 [2.5–3.8]	3.4 [2.7–4.2]	**0.013**
	81 (11.8)	68 (11.5)	13 (13.5)	0.558
Serum folate (ng/mL) (n = 606), median [IQR] < 3.3 (ng/mL)	5.1 [3.4–7.7]	5.1 [3.4–7.6]	6.0 [3.1–9.5]	0.249
	146 (24.1)	122 (23.5)	24 (27.6)	0.41
Urates (mg/dL), median [IQR] > 7.2 mg/dL men; > 6 mg/dL women	5.2 [4.3–6.3]	5.1 [4.2–6.1]	5.9 [5–7.3]	** < 0.001**
	108 (14.8)	76 (12.1)	32 (31.7)	** < 0.001**
Albumin (g/L) (n = 718), median [IQR] < 35 g/L	39.9 [36.8–42.3]	39.8 [36.7–42]	40 [37.8–43.4]	0.205
	103 (14.3)	91 (14.7)	12 (12.0)	0.471
Creatinine (mg/dL) (n = 718), median [IQR] > 1.2 mg/dL	0.79 [0.68–0.92]	0.79 [0.68–0.9]	0.83 [0.69–0.97]	**0.021**
	13 (1.8)	6 (1.0)	7 (6.9)	** < 0.001**
Total bilirubin (mg/dL) (n = 718), median [IQR] > 1.2 mg/dL	0.70 [0.49–1.11]	0.7 [0.5–1.1]	0.6 [0.5–1.0]	0.346
	153 (21.3)	136 (22.0)	17 (17.0)	0.257
eGFR (mL/min/1.73 m^2^) (n = 724), median [IQR] ≥ 90 mL 60–90 mL < 60 mL	105.5 [90.4–123.3]	106.4 [91.7–123.7]	94.8 [82.3–118.8]	**0.001**
	545 (75.3)	485 (77.8)	60 (59.4)	** < 0.001**
	165 (22.8)	131 (21.0)	34 (33.7)	
	14 (1.9)	7 (1.1)	7 (6.9)	
AST (U/L), median [IQR] > 37 U/L	38 [22–72.3]	27.8 [21.6–74]	41 [24–70]	0.678
	375 (51.5)	319 (50.9)	56 (55.4)	0.394
ALT (U/L) (n = 724), median [IQR] > 41 U/L	32 [18–55.5]	31 [17.4–54]	36 [23–61]	**0.017**
	274 (37.8)	229 (36.8)	45 (44.5)	0.134
AST/ALT (n = 724), median [IQR] > 2	1.2 [0.9–1.7]	1.2 [0.9–1.7]	1 [0.7–1.5]	** < 0.001**
	115 (15.8)	102 (16.4)	13 (12.9)	0.372
GGT (U/L) (n = 720), median [IQR] > 50 U/L	120.8 [49.9–325.5]	116 [43.8–317]	134.4 [75–435]	**0.027**
	536 (74.4)	445 (71.9)	91 (90.1)	** < 0.001**

ESR: erythrocyte sedimentation rate; MCV: mean corpuscular volume; eGFR: estimated glomerular filtration rate; AST: aspartate aminotransferase, ALT: alanine aminotransferase; GGT: gamma-glutamyl transferase. Significance values are in Bold.

Regarding pre-existing medical co-morbidity, 26.7%, 9.2%, and 6.8% of the patients had a history of hypertension, type 2 diabetes, and CVD, respectively. Overall, the median BMI at admission was 25.0 kg/m^2^ (IQR: 22.1–28.7 kg/m^2^).

Prevalence of hypertriglyceridemia, hyperglycemia and low HDL cholesterol was 33.2%, 27.1% and 20.1%, respectively.

### Prevalence and characteristics of MetS patients

The prevalence of MetS was 13.9% (101/728) with no differences between men and women. Among the patients with MetS, 27.7% had a history of type 2 diabetes, and 78.2% showed elevated fasting glucose levels at admission. Furthermore, 86.9% of MetS patients had obesity, 70.3% had hypertension, 15.3% had a history of CVD, 80.2% had high triglycerides, and 67.3% had low HDL cholesterol (50% in men and 66.7% in women). The prevalence of atherogenic dyslipidemia among those with MetS was 70.2%.

### Associations of MetS

MetS patients were older (51 years vs. 46 years, *P* < 0.001) as compared to those without MetS (Table 1).

**Figure 1:**
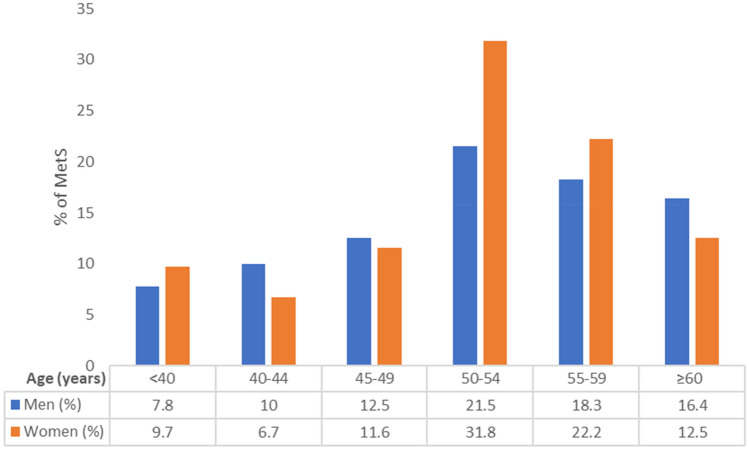
Prevalence of MetS in 728 patients with alcohol abuse or dependence by sex and age groups.

Figure 1 shows the prevalence of MetS by sex and age groups (< 40 years, 40–44 years, 45–49 years, 50–54 years, 55–59 years, and ≥ 60 years). Prevalence of MetS in men significantly increased from 7.8% in the youngest age group (< 40 years) to 21.5% in the 50–54 years age group (*P* = 0.013) and decreased in those older than 55 years (18.3% and 16.4% in the age group of 55–59 years and ≥ 60 years, respectively). In women, prevalence of MetS significantly increased from 9.7% in the youngest age group (< 40 years) to 31.8% in the 50–54 years age group (*P* = 0.045) and decreased to 22.2% and 12.5% in the age groups of 55–59 years and ≥ 60 years, respectively. There were no statistically significant differences in the overall prevalence of MetS by sex and age group.

Univariate analysis (Table 1) showed that patients with MetS had higher prevalence of hyperuricemia (31.7% vs. 12.1%, *P* < 0.001), higher prevalence of serum creatinine > 1.2 mg/dL (6.9% vs. 1.0%, *P* < 0.001) and higher prevalence of GGT > 50 U/L (90.1% vs. 71.9%, *P* < 0.001). In addition, there was an association between eGFR and MetS: prevalence of MetS was 11.0%, 20.6%, and 50.0% for eGFR > 90 mL/min, eGFR between 60 and 90 mL/min, and eGFR < 60 mL/min, respectively (*P* < 0.001).

In multivariate analysis, reduced eGFR, hyperuricemia, serum GGT > 50 U/L and age at admission were independently associated with MetS (Table 2). Specifically, there was a statistically significant, dose–response effect between the eGFR and MetS; relative to patients with eGFR > 90 mL/min, those with eGFR between 60–90 mL/min and those with eGFR < 60 mL/min were 1.93 (95% confidence interval [CI] 1.18–3.15) and 5.61 (95% CI 1.66–19.0) times more likely to have MetS, respectively. Furthermore, the odds of MetS were significantly higher in patients with hyperuricemia (OR 2.28, 95% CI 1.36–3.82) as compared to those with normal SUA levels, as well as in those with serum GGT > 50 U/L (OR 3.67, 95% CI 1.80–7.46) as compared to those with normal serum GGT levels. In addition, MetS was significantly associated with age (OR 1.03; 95% CI 1.01–1.05, for every increase of 1 year in age).

**Table 2:** Logistic regression model for the associations of MetS in 728 patients with alcohol abuse or dependence admitted for detoxification.

	Univariate OR (95% CI)	*P* value	Multivariate** aOR (95% CI)	*P* value
Age: increase of 1 year	1.04 (1.02–1.06)	< 0.001	1.03 (1.01–1.05)	0.013
eGFR (mL/min)				
> 90	1		1	
60–90	2.10 (1.32–3.33)	0.002	1.93 (1.18–3.15)	0.008
< 60	8.08 (2.74–23.8)	< 0.001	5.61 (1.66–19.0)	0.006
GGT > 50 (U/L)	3.56 (1.81–6.99)	< 0.001	3.67 (1.80–7.46)	< 0.001
Hyperuricemia*	3.36 (2.07–5.45)	< 0.001	2.28 (1.36–3.82)	0.002

*Urate > 7.2 mg/dL in men and > 6 mg dL in women.

** adjusted by age, eGFR, GGT > 50 and hyperuricemia.

eGFR: estimated glomerular filtration rate; GGT: gamma-glutamyl transferase; OR: odds ratio.

The regression model showed adequate fit as measured by the Hosmer–Lemeshow statistic (χ^2^ = 182.1, *P* = 0.974).

### Sensitivity analysis

A sensitivity analysis taking into account BMI values > 25 kg/m^2^ showed a MetS prevalence of 20.5%, and the associations with MetS were consistent with those presented in the main analysis.

## Discussion

Behind smoking and obesity, excessive alcohol consumption is the third leading cause of premature death in western countries. However, the effect of harmful drinking on the risk of MetS is partially known. This study in middle-aged patients starting a treatment for alcohol abuse or dependence shows that prevalence of MetS is lower than that reported in the limited literature^13^. The relatively low prevalence of MetS in this large series might be related to the observed proportion of heavy drinkers without a history of cardiometabolic disease (i.e., coronary artery disease, type 2 diabetes) thus suggesting that the majority of cases were premorbid for MetS. Interestingly, prevalence of premorbid MetS in the Spanish general population is around 17%^21^ and mainly related to both overweight/obesity and hypertension. Moreover, prevalence of MetS may vary depending on the case definition, selection of patients, age, sex, ethnicity, and geographic area^22^. In this regard, the only systematic review and meta-analysis of alcohol-related MetS was published in 2016, which included five studies and an estimated mean prevalence of 19.3% (95% CI 14.3–25.6)^13^; four of the five studies analyzed less than 200 patients without reporting associations of MetS and the remaining focused on the risk factors.

Our results in this series of apparently asymptomatic heavy drinkers primarily admitted for the treatment of the disorder indicate that renal function plays a role in the alcohol-related MetS. Furthermore, the significant dose–response effect of declining renal function on MetS is observed in those with mild renal impairment. Relative to those with normal eGFR, patients with eGFR < 60 mL/min were more than five times more likely to meet criteria of MetS. The MetS can increase the risk of kidney disease via mechanisms linked to chronic inflammation and oxidative stress^23^. Moreover, type 2 diabetes and hypertension have been extensively associated with impaired kidney function, and although least understood, it should be noted that obesity has been recently correlated with lower eGFR and chronic kidney disease^24^. Nevertheless, continuous health interventions involving regular monitoring of BMI and lifestyle changes have led to improved renal function outcomes^25,26^. Interestingly, a population-based longitudinal study concluded that individuals with MetS are at a higher risk of developing kidney disease, although the risk is reduced or disappears when MetS is reversed^27^. To the best of our knowledge, this is the first study that shows the association of kidney disease and MetS in heavy drinkers. In the only meta-analysis on MetS in AUD patients none of the five studies analyzed the role of kidney disease in the syndrome^13^. Besides, some other studies in different populations at risk of MetS included the alcohol consumption as a covariate but they did not analyze the association of eGFR and MetS^28–30^.

Regarding the role of serum GGT, its association with MetS has been described in general population and cohort studies^31,32^. Elevated serum GGT is a marker of intracellular liver triglyceride accumulation that has been associated with overweight/obesity and insulin resistance, regardless of alcohol consumption^31,33^.

Moreover, elevated serum GGT may reflect the impact of oxidative stress and chronic inflammation and both conditions are closely associated with MetS^34,35^. In fact, serum GGT is an early marker for atherosclerosis, arterial stiffness and plaque^36^.

Hyperuricemia has been associated with MetS and coronary artery disease regardless of alcohol consumption^37,38^. As for the association between SUA and MetS, a recent study by our group described a close relationship between hyperuricemia and serum GGT in AUD patients^15^. Furthermore, individuals with elevated SUA levels are at risk of developing chronic renal dysfunction according to a systematic review including 13 studies and 190.000 participants^39^.

Epidemiologic observations have suggested an independent association between age and MetS. Prevalence of MetS increases up to the sixth decade of life in men and the seventh decade in women and then decline^40,41^. Our results show that prevalence of MetS starts to decrease a decade earlier as compared to the general population thus pointing out the need for future research in individuals with an alcohol-induced pro-inflammatory environment^42^.

This study had several limitations that must be mentioned. First, the cross-sectional design did not support conclusions regarding the causality of the findings. Moreover, in this case-series there were no variables related to socioeconomic status and lifestyle, such as diet and physical activity and both conditions have been associated with MetS^43^. Furthermore, this study lacked indicators of psychiatric comorbidity and use of antipsychotic drugs that might influence the appearance of metabolic side effects including overweight/obesity^13^. Finally, the results of this study focused only on patients with alcohol abuse or dependence, and they cannot be generalized to individuals with moderate alcohol consumption. However, the main strength of this study is the number of behavioral and biological variables to analyze associations of MetS in a poorly studied population.

In conclusion, alterations in eGFR, serum GGT, and SUA were independently associated with MetS in AUD patients which suggests that screening of renal impairment, liver damage and metabolic alterations could be of interest in the clinical evaluation of heavy drinkers.
